# Reverse Linear Neuro Periodization Model for Rehabilitation After Arthroscopic Rotator Cuff Repair: A Narrative Review

**DOI:** 10.3390/clinpract15060105

**Published:** 2025-05-30

**Authors:** Georgios Kakavas, Emmanouil Brilakis, Maria Papatzikou, Nikolaos Malliaropoulos, Jean Mazeas, Florian Forelli

**Affiliations:** 1 Fysiotek Spine & Sports Lab, 17562 Athens, Greece; georgios.kakavas@gmail.com; 2 Brilakis Orthopaedics, 17561 Palaio Faliro, Greece; emmanuel.brilakis@gmail.com (E.B.); m.papatzikou@gmail.com (M.P.); 3 Department of HYGEIA General Hospital, 15123 Athens, Greece; 4 Thessaloniki Sports Medicine Clinic, 54622 Thessaloniki, Greece; contact@sportsmed.gr; 5 Sports Clinic, Rheumatology Department, Barts Health NHS Trust, London E1 1BB, UK; 6 Orthosport Rehab Center, 95330 Domont, France; jeanmazeas@gmail.com; 7 Orthopedic and Surgery Department, @OrthoLab, Clinic of Domont, 95330 Domont, France; 8 SFMKS-Lab, Société Française des Masseurs-Kinésithérapeutes du Sport, 93380 Pierrefitte sur Seine, France; 9 Haute-Ecole Arc Santé, HES-SO University of Applied Sciences and Arts Western Switzerland, 2800 Delémont, Switzerland

**Keywords:** periodization, rehabilitation, neurocognitive, shoulder, rotator cuff

## Abstract

Periodization is a concept of systematic progression in training and rehabilitation. The rehabilitation literature, however, is scarce, with information about optimally designing resistance training programs based on periodization principles for injured athletes. This periodization model—reverse linear neuro periodization—is a model proposed for the long-term rehabilitation needed after an arthroscopic rotator cuff repair. With recent evidence supporting neural contributions to shoulder injuries and the rate of recovery, rehabilitation protocols may benefit from incorporating approaches that target the sensorimotor system. Integrating motor learning principles (external focus and differential learning) and new technologies (virtual reality, laser pointers, stroboscopic glasses) may bolster current shoulder rehabilitation protocols and improve patient recovery times and outcomes. Such an understanding allows well-informed sport rehabilitation specialists to better bridge the gap between the preparation for competition widely used by coaches and the treatment of injuries that may occur.

## 1. Introduction

Rotator cuff pathology is one of the most prevalent causes of shoulder pain and dysfunction, particularly in individuals over 40, with studies estimating a prevalence of 20–30% in the general population and over 60% in individuals older than 60 [1]. Symptomatic tears can result in long-term disability, reduced quality of life, and significant health service utilization [2]. Despite advancements in surgical techniques, many patients continue to experience functional limitations post-surgery [3], underscoring the importance of optimized rehabilitation strategies [4]. Current clinical guidelines often lack individualized neurocognitive components, which are critical given the evidence of central nervous system involvement in musculoskeletal injuries [5], and the emerging role of sensorimotor training in rehabilitation [6]. Moreover, rotator cuff disease is a leading indication for shoulder surgery, with arthroscopic repair procedures increasing significantly over the past two decades [4,7]. These factors highlight the urgent need for structured, effective, and individualized rehabilitation strategies to optimize postoperative outcomes and reduce re-injury risk [8,9].

Recent advancements in arthroscopic techniques and a deeper understanding of rotator cuff healing must be integrated with effective rehabilitation strategies following arthroscopic rotator cuff repair [10]. Developing tailored rehabilitation programs for both elite athletes and recreational participants aiming to maximize recovery and enhance performance is a complex task [11]. Periodization, which refers to the structured progression of training, is particularly useful in this context. Specifically, resistance training programs are designed to follow predictable changes in key training variables [4,12]. While there is extensive research on strength training, particularly regarding different periodization models for healthy athletes, there is a lack of comprehensive studies addressing how to optimally apply these principles in rehabilitation for injured athletes. Longo et al., in their two-year follow-up study, found no significant improvement in shoulder function in terms of central nervous system performance [5]. Further high-quality randomized controlled trials with longer-term follow-ups are needed to determine if surgical and conservative treatments yield comparable long-term outcomes.

The reverse linear neuro periodization model offers a structured framework specifically designed for postoperative shoulder rehabilitation. Conventional periodization typically progresses from high training volume and low intensity to lower volume and higher intensity to peak physical performance. In contrast, the reverse linear neuro periodization model begins with low intensity and high volume, aligning with early-stage rehabilitation needs and promoting neuromuscular adaptation while minimizing stress on healing tissues. This approach focuses on the sensorimotor system, which plays a crucial role in joint stability and movement coordination—areas often compromised after surgery [13]. Additionally, incorporating neurocognitive rehabilitation exercises, such as virtual reality and stroboscopic glasses, boosts cognitive involvement, enhancing communication between the brain and body, and potentially speeding up recovery [14]. This flexible, multi-dimensional method aligns the rehabilitation intensity with the body’s natural healing process for optimal results.

The sensorimotor system encompasses all components, efferent, and central integration that are involved in maintaining joint stability during movement. Dysfunction in this system can lead to joint damage, a common consequence of ligament injuries [15]. Strength training is one of the most important elements in any rehabilitation program. Effective programs include components such as endurance, flexibility, proprioception, balance, joint mobility, and power [6]. One major challenge in creating these programs is designing training regimens that promote both neuro and muscular adaptations while considering the athlete’s biological healing processes and ensuring safety [16]. Much of the existing strength training research involves healthy, trained, and untrained individuals [17]. Additionally, ongoing monitoring of athletes throughout rehabilitation remains a significant challenge.

Traditional rehabilitation protocols following arthroscopic rotator cuff repair are primarily focused on restoring joint mobility and muscle strength through biomechanical exercises [12,18,19]. However, these approaches often overlook essential components such as individualized progression, neurocognitive engagement, and sensorimotor control. As a result, patients may experience prolonged recovery times, persistent coordination deficits, and a heightened risk of re-injury [20,21,22]. To address these limitations, we propose a novel model—reverse linear neuro periodization—that integrates evidence-based training progression with targeted cognitive and sensorimotor stimulation strategies. This model offers a more comprehensive and patient-centered framework for rehabilitation, bridging the gap between traditional physical therapy and the complex demands of functional and athletic performance.

This narrative review aims to discuss relevant training variables and methods of reverse linear periodization, as well as periodization rehabilitation outcomes. A secondary purpose is to provide an anecdotal framework for implementing reverse linear periodization principles into the postoperative rehabilitation programs following an arthroscopic rotator cuff repair.

## 2. Methodology for Conducting the Narrative Review

This narrative review was conducted to provide a comprehensive synthesis of the existing literature on rehabilitation following arthroscopic rotator cuff repair, with a specific focus on periodization models and the introduction of the reverse linear neuro periodization model.

To identify relevant studies, we performed a systematic search in the following electronic databases: PubMed, Scopus, Web of Science, and Google Scholar. The search was conducted using a combination of keywords, including “rotator cuff repair”, “rehabilitation”, “periodization”, “reverse periodization”, and “shoulder surgery recovery”. The inclusion criteria were: studies published in peer-reviewed journals, articles focusing on rehabilitation strategies and protocols after rotator cuff repair, research articles, reviews, and guidelines published in English. The exclusion criteria were studies unrelated to rotator cuff repair or postoperative rehabilitation, articles not available in full text, publications in languages other than English.

We also reviewed the references of included articles to identify additional relevant studies. The data extracted from the selected studies were synthesized to highlight key rehabilitation concepts and provide a rationale for the introduction of the reverse linear neuro periodization model.

This narrative review does not aim to provide a systematic evaluation of the literature but rather to contextualize and integrate the findings to propose a novel approach to postoperative rehabilitation. However, we adhered to key methodological principles inspired by the Preferred Reporting Items for Systematic Reviews and Meta-Analyses (PRISMA) guidelines to enhance transparency and scientific rigor. While we did not perform a formal risk of bias assessment, the methodological quality of the included articles was considered by selecting peer-reviewed publications with clearly defined objectives, robust study designs, and relevance to the topic. Systematic reviews, clinical guidelines, and original research with explicit methodologies were prioritized to support the credibility of the synthesized findings.

## 3. Postoperative Rehabilitation Periodization Models

There is a limited amount of research on the application of periodization models within rehabilitation, despite their potential utility. Nevertheless, several models for advancing athletes through training can be effectively adapted for rehabilitation purposes. Periodization is a systematic approach used by clinicians to design resistance training programs [23]. It involves the deliberate adjustment of training variables such as load, sets, repetitions, and mental fatigue to maximize training adaptations and prevent overtraining syndrome [7,8]. Although periodization is generally necessary for achieving maximal strength gains, there is some opposing evidence [24]. The concept of periodization is rooted in Selye’s general adaptation syndrome, which posits that systems adapt to stressors in order to meet the demands they face [9]. The aim of a periodized program is to optimize the overload principle, which is the process through which the neuromuscular system adapts to new loads or stressors [23,24]. A periodized training plan specifies variables like intensity, volume, and frequency, and their interaction leads to the overload necessary for adaptation [25,26]. If the system adapts to these stressors without a corresponding increase in overload, further progress may be halted [10]. Periodization prevents this issue by continuously varying the neuromuscular load. Although several periodization models exist, two primary approaches are most common. The first is the traditional linear model, which involves gradually adjusting exercise volume and load across several planned mesocycles [25,27,28]. The macrocycle covers the entire rehabilitation period from the first postoperative day to full return to sport, with subdivisions called mesocycles and microcycles [25,27,28]. The duration of each cycle depends on the individual patient’s progress, rather than adhering to rigid numerical guidelines. Nonlinear periodization, which has gained favor over linear periodization, involves more frequent changes in volume and load, typically on a daily, weekly, or biweekly basis, to provide the neuromuscular system with more frequent recovery opportunities [11]. Shorter phases with more frequent stimuli changes may enhance strength gains. Kraemer and Fleck expanded on this by introducing both planned and flexible nonlinear periodization [12]. This model incorporates a predicted loading scheme but allows for adjustments based on the athlete’s condition. Finally, reverse linear periodization (RLP) differs from traditional models by modifying load and volume in the opposite direction: increasing volume and reducing load [13,14]. Periodization can be achieved through various adjustments, such as modifying sets, repetitions, exercise order, the number of exercises, resistance, rest intervals, contraction types, and training frequency, offering a wide range of periodization options [15]. To date, no direct comparative studies have evaluated the reverse linear neuro periodization model against traditional or nonlinear rehabilitation frameworks in the context of rotator cuff repair. However, the model is theoretically supported by principles of neuroplasticity, sensorimotor integration, and adaptive load management derived from evidence in related domains such as ACL rehabilitation and athletic training. Future research is needed to empirically assess its effectiveness and validate its potential superiority over conventional approaches.

## 4. Neurocognitive Approach in Postoperative Rotator Cuff Repair Rehabilitation

The neurocognitive rehabilitation component within the reverse linear neuro periodization model is a critical and innovative element designed to enhance shoulder recovery by actively engaging the central nervous system alongside traditional physical exercises. This approach integrates cognitive, sensory, and motor elements at each phase of rehabilitation, aiming to rebuild neural pathways that are essential for complex movements, proprioception, and overall motor control. Unlike traditional rehabilitation that focuses primarily on strength and flexibility, neurocognitive rehabilitation addresses deficits in the brain’s ability to process and coordinate sensory and motor signals—a common issue following surgery that can lead to instability, compensatory movement patterns, and increased risk of re-injury [5,6].

In the immediate postoperative phase, when the shoulder is immobilized and direct movement is restricted, neurocognitive rehabilitation can begin with exercises that involve mental visualization and cognitive interference tasks. These exercises encourage the patient to mentally engage with movement patterns, practicing shoulder mobilization in the mind to initiate neural activation and motor planning. For instance, patients may use guided imagery techniques to imagine shoulder movements or perform “virtual” repetitions of exercises they will eventually conduct physically. Additionally, cognitive interference tasks, such as responding to auditory or visual cues while mentally focusing on shoulder stabilization, can further stimulate brain areas involved in motor control, preparing the nervous system for future physical engagement [6,29].

As the patient transitions to the active assisted and strengthening phases, neurocognitive tools like stroboscopic glasses and laser pointer exercises are introduced to add controlled visual and cognitive challenges. Stroboscopic glasses intermittently block vision, forcing the brain to rely on proprioceptive and tactile feedback to maintain coordination. For example, a patient wearing stroboscopic glasses may perform basic shoulder exercises, such as reaching movements or object manipulation, where they must react to visual disruptions, thereby enhancing reaction time, focus, and adaptive proprioceptive responses. Similarly, laser pointer exercises, where patients are asked to trace shapes or aim at specific points on a wall using their shoulder movements, require precise control and steady movement, reinforcing sensorimotor accuracy and stability [6,11].

In the advanced strengthening and return-to-sport phases, neurocognitive rehabilitation becomes more dynamic and sport-specific. Here, immersive virtual reality (VR) environments and dual-task training are incorporated to simulate the cognitive and physical demands of real-life activities or athletic movements. For instance, VR scenarios might place the patient in a virtual sports setting—such as blocking an oncoming ball in a simulated game—requiring rapid reaction times, hand-eye coordination, and proprioceptive awareness. This immersive environment provides the brain with complex stimuli, fostering neural adaptations that closely resemble the challenges encountered in sports or daily life. Additionally, dual-task training, where patients perform motor tasks (like catching a ball) while solving simple mental tasks (such as basic math or reaction tests), further strengthens their ability to manage multiple stimuli, a skill that is crucial for return-to-sport readiness [6,30].

This phase-specific neurocognitive approach not only accelerates physical recovery but also builds mental agility and neural resilience, reducing the likelihood of re-injury and improving functional outcomes. By progressively challenging the sensorimotor system with adaptable exercises, this rehabilitation model empowers patients to rebuild confidence and ensure stability across all movement ranges, ultimately preparing them for the physical and cognitive demands of both everyday activities and high-level athletic performance. Neurocognitive rehabilitation, therefore, serves as a vital component within the overall recovery framework, bridging the gap between physical therapy and the neurological demands of real-world movement [23].

To enable objective monitoring of the neurocognitive component of rehabilitation, we outline here a concise set of metrics that can be seamlessly incorporated into clinic or research workflows. Visuomotor processing speed can be tracked with computer-based reaction-time tasks or light-board latency (e.g., FitLight^®^), while proprioceptive acuity is quantifiable through joint-position-sense error—either laser-pointer trace deviation or active angle-reproduction tests. Cognitive-motor integration is best captured by the dual-task cost, calculated as the percentage performance decrement when a functional movement (reach, catch, closed kinetic chain upper extremity stability test, Upper-Quarter Y-Balance) is performed concurrently with a cognitive load. Finally, pairing these objective measures with patient-reported questionnaires that probe perceived neurocognitive confidence ensures a comprehensive appraisal of both physiological and experiential outcomes, thereby providing a robust framework for empirically validating the benefits of the proposed neurocognitive techniques.

Despite their promise, neurocognitive rehabilitation technologies face several pragmatic hurdles that could limit broad adoption across clinical environments. Upfront costs for specialized hardware (e.g., immersive virtual reality headsets, stroboscopic glasses, and light-board systems) and ongoing maintenance may exceed the budgets of smaller clinics, while limited floor space, network bandwidth, and infection-control protocols can further constrain deployment. Successful use also depends on staff expertise; therapists need dedicated training time to become proficient in calibrating devices and interpreting data, which may not be reimbursed. In populations with low digital literacy, patient engagement and compliance can suffer, and strict data-privacy regulations may complicate cloud-based analytics. To mitigate these barriers, we propose a phased implementation strategy that prioritizes low-cost or already-available platforms (e.g., tablet-based cognitive-motor apps and laser-pointer tasks), integrates brief, modular staff workshops, and leverages tele-rehabilitation to extend reach without additional physical space. Advocacy for reimbursement codes specific to technology-assisted neurorehabilitation and collaboration with academic or industry partners to provide shared equipment pools can further enhance feasibility and sustainability.

## 5. Application of Reverse Linear Neuro Periodization Model in Postoperative Rotator Cuff Repair Rehabilitation

Rehabilitation programs typically follow a basic progressive overload model, focusing primarily on the injured area. Periodized training, which is a safe approach for both older adults and individuals in pain, offers substantial benefits [22]. However, specific guidelines for adjusting resistance training variables in rehabilitation protocols are often missing. Clinicians tend to have general goals for each phase, along with broad precautions and a somewhat incomplete list of exercises. Various studies have compared eccentric training to standard rehabilitation methods following ACL reconstruction and examined the differences between open versus closed-chain exercises for ACL-deficient knee rehabilitation and patellofemoral pain, as well as home versus supervised physical therapy protocols [23]. In addition to comparing linear and nonlinear periodization models within the framework of standard rehabilitation protocols, researchers could explore the effects of different training modalities or the duration of training. By employing both linear and nonlinear periodization as a framework, rotator cuff repair patients could be divided into three groups: control, linear, and nonlinear [24]. Traditional rehabilitation could also be compared to eccentric-focused programs, or eccentric exercises could be integrated into periodization models.

For patients who have undergone arthroscopic rotator cuff repair, rehabilitation can be tailored with endurance, hypertrophy, strength, and power-focused days, depending on the timeline, using a 3-day-per-week program [16,18]. As the athlete progresses to more sport-specific drills such as plyometrics and agility in the later stages, emphasis can shift to strength, power, and hypertrophy training. During the final stages of rehabilitation, power, strength, or hypertrophy may be prioritized due to the athlete’s existing deficits before returning to sport [17]. Hypertrophy training can be particularly beneficial for athletes with ongoing deficits in rotator cuff muscles. Postoperative shoulder rehabilitation is a complex, multi-faceted process that must continuously adapt to meet the physiological and psychological needs of the individual athlete [21]. The sensorimotor system plays a crucial role in maintaining joint stability during movement. After ACL injuries and reconstructions, sensorimotor deficits, such as altered proprioception and changes in neuromuscular control, can occur. The impact of the type of autograft used on knee sensorimotor function remains unclear. The emphasis between recovery and adaptation will depend on the specific needs of the athlete in the context of their procedure. This highlights the importance of employing rehabilitation strategies that are periodized to mirror the demands of sport while also promoting recovery and adaptive responses [16,20]. Postoperative rehabilitation should move beyond traditional biomechanics and muscle strength approaches and address the subtle sensorimotor control deficits that are critical for full recovery and readiness for both everyday activities and sports performance [25].

Because patient recovery after rotator-cuff repair is inherently heterogeneous, we recommend using a criteria-based rather than time-based progression in which each phase’s exercises are advanced only when the individual meets clearly defined thresholds: (1) pain ≤ 2/10 on a numeric scale during and 24 h after activity; (2) passive and active range of motion (ROM) within ±10% of the contralateral side for the movements required in the next phase; (3) isometric rotator-cuff strength ≥ 50% body-weight–adjusted norm before introducing external load; and (4) dual-task cost < 20% on a chosen proprioceptive test (e.g., Upper-Quarter Y-Balance) before adding higher-level neurocognitive drills. Clinicians should also adjust weekly training volume (sets × reps) and neurocognitive load (stimulus density, reaction-time targets) by ±10–20% in response to objective fatigue markers (sleep quality, rate perceived exertion) and any flare-up in inflammation or pain. For older adults or patients with comorbidities, priority can shift to slower tempo, lower-amplitude movements and shorter cognitive-challenge bouts, whereas competitive athletes may progress more rapidly by substituting higher-velocity plyometrics and sport-specific virtual reality scenarios once phase criteria are met. This criteria-driven, patient-specific modulation ensures safety while preserving the individualization that optimizes neuromuscular and neurocognitive adaptation.

## 6. Clinical Practices of Reverse Linear Neuro Periodization Model in Postoperative Rotator Cuff Repair Rehabilitation

This section provides clinicians with detailed, phase-specific examples for implementing the reverse linear neuro periodization model in postoperative shoulder rehabilitation. These examples ensure that each stage of recovery is safely tailored to the patient’s needs, supporting neuromuscular adaptation, stability, and progressive strengthening.

### 6.1. Phase 1: Immediate Postoperative Phase (Weeks 1–2)

During this phase, protecting the repaired tendon and reducing pain and inflammation are top priorities. Exercises are passive and low-intensity to avoid stressing the repair, but neurocognitive engagement is introduced to initiate mental and neural activation [3,4,21,31,32,33,34,35,36,37] (Table 1).

- Passive Range of Motion (ROM): Gentle passive movements, such as pendulums and assisted shoulder elevation, can prevent stiffness and maintain initial joint mobility. Clinicians should guide patients to perform pendulum exercises by letting the arm hang and gently swinging it in small circular motions, ensuring no active muscle engagement around the shoulder.
- Mental Visualization and Cognitive Interference Tasks: Neurocognitive engagement can start with guided mental visualization. Patients visualize moving their shoulder in specific patterns, such as flexion or rotation, engaging motor planning areas in the brain. Cognitive interference tasks, like responding to sounds while mentally focusing on shoulder stability, can reinforce neural connections. For example, a clinician may ask the patient to focus on shoulder stabilization while listening for specific auditory cues, initiating early neural adaptation.

### 6.2. Criteria to Progress to Phase 2

- Pain and inflammation are controlled or significantly reduced.
- The patient demonstrates the ability to perform passive range-of-motion exercises (e.g., pendulum exercises) without exacerbating symptoms.
- No signs of complications, such as infection or increased swelling.
- Maintenance of joint integrity without signs of repair compromise.

### 6.3. Phase 2: Intermediate Postoperative Phase (Weeks 3–6)

This phase aims to gradually introduce active motion through controlled, pain-free exercises. Neurocognitive tools begin to incorporate visual and proprioceptive challenges to encourage coordination and reaction adaptation [3,4,21,32,33,34,35,36,37] (Table 2).

**Figure 1:**
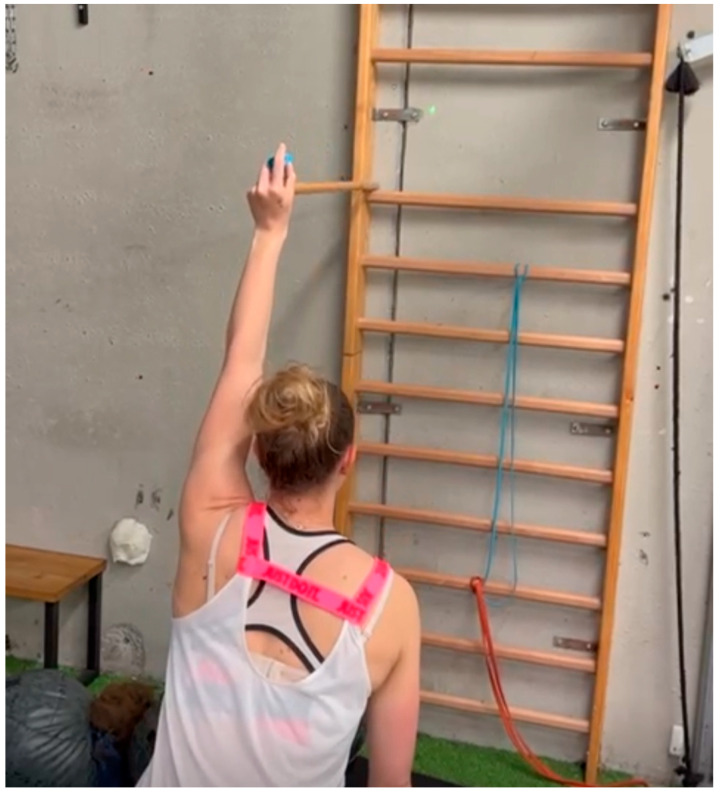
Laser pointer exercises for proprioception.

- Active-Assisted Movements: Active-assisted exercises, like wall slides, involve the patient using their non-affected arm or a therapist’s assistance to guide the shoulder through gentle motions without full muscle engagement. Wall slides encourage patients to slowly slide their hand up a wall, promoting gradual shoulder elevation in a controlled manner.
- Laser Pointer Exercises for Proprioception: Clinicians can attach a laser pointer to the patient’s hand and ask them to trace shapes or follow a target on the wall. For example, the patient can practice drawing circles or squares with the laser, enhancing their spatial awareness, control, and proprioceptive feedback as they refine movements (Figure 1).
- Stroboscopic Glasses for Reaction and Balance: Stroboscopic glasses intermittently block vision, forcing the patient to rely on proprioceptive feedback. Patients wearing these glasses can perform seated or standing shoulder exercises, like shoulder flexion or holding a position against light resistance, while managing the visual disruption, and improving sensory processing and coordination.

### 6.4. Criteria to Progress to Phase 3

- The patient demonstrates tolerance for active-assisted movements with minimal or no pain.
- Improved proprioception and neuromuscular control during basic tasks (e.g., wall slides and laser pointer exercises).
- Full passive range of motion achieved within safe limits for the surgical repair.
- No swelling or irritation following exercises, indicating stability of healing tissue.

### 6.5. Phase 3: Active Range of Motion and Strengthening Phase (Weeks 6–12)

At this stage, active movement and light strengthening exercises support muscle engagement around the shoulder while progressively improving the range of motion and stability [3,4,21,32,33,34,35,36,37] (Table 3).

**Figure 2:**
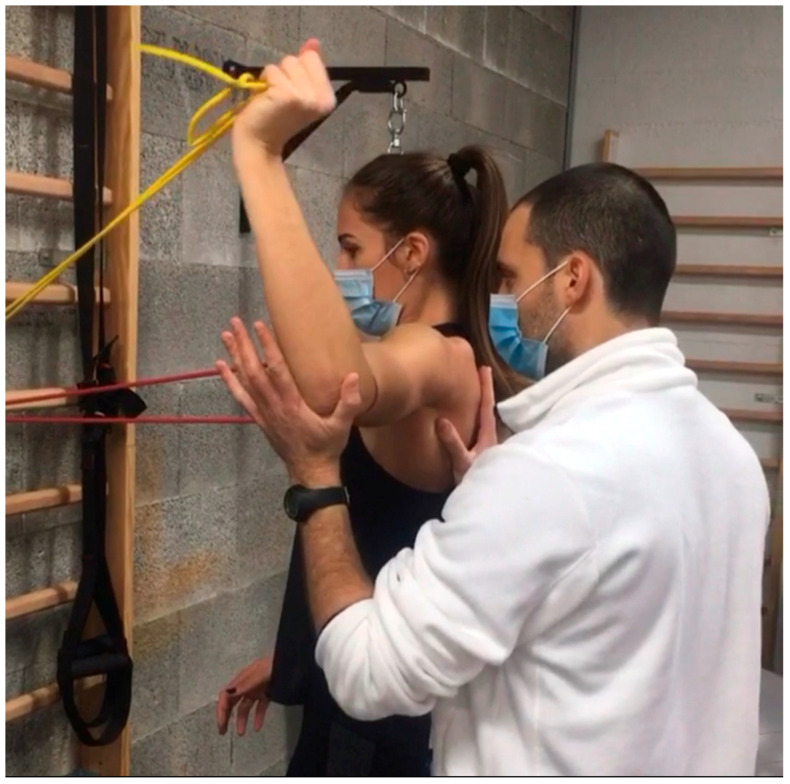
Resistance band exercises.

- Active Shoulder Elevation and Rotation: Patients begin active movements such as shoulder elevation, external rotation, and gentle abduction within a pain-free range. Exercises are typically performed in a supported or supine position initially (e.g., lying down to avoid gravity’s impact), then progressing to seated or standing as tolerance improves.
- Resistance Band Exercises: Introducing low-resistance bands provides safe, gradual strengthening. Patients can perform seated rows, external rotations, or internal rotations with light bands, allowing for incremental load. For instance, a clinician might start with a resistance band around the hands, guiding the patient through a controlled external rotation against mild resistance to strengthen stabilizing muscles (Figure 2).
- VR-Based Neurocognitive Drills: Virtual reality can simulate interactive environments that promote shoulder engagement while challenging reaction times and hand–eye coordination. For example, a VR scenario could involve a virtual game where patients use small shoulder movements to “catch” or “block” objects on the screen, requiring accurate control and reaction to dynamic stimuli.

### 6.6. Criteria to Progress to Phase 4

- The patient achieves a full active range of motion without compensatory movements or pain.
- Ability to perform light resistance exercises (e.g., resistance bands) with proper form and control.
- Demonstrates stable shoulder movement during VR drills or other proprioceptive tasks.
- Improved functional use of the shoulder in daily activities without discomfort.

### 6.7. Phase 4: Initial Strengthening Phase (Weeks 12–18)

This phase focuses on progressively increasing shoulder strength and endurance while emphasizing functional, controlled movements that prepare the patient for daily activities and light sports [3,4,21,32,33,34,35,36,37] (Table 4).

**Figure 3:**
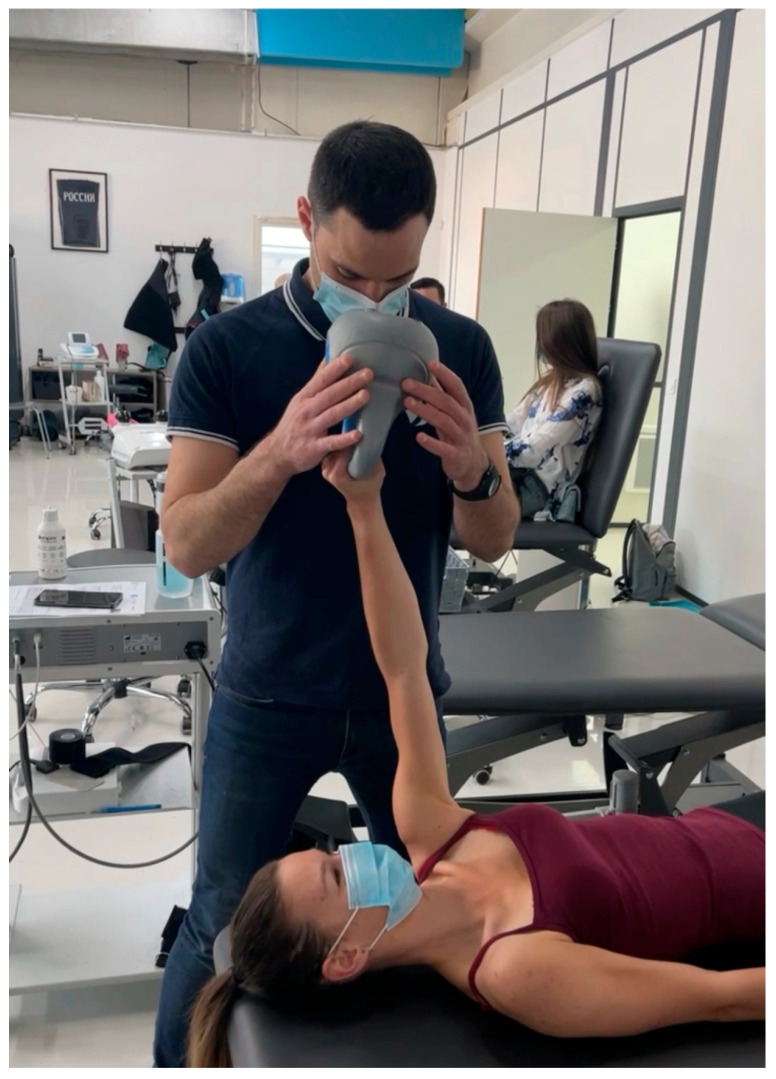
Perturbation training.

- Strengthening with Resistance: More resistance is gradually added through exercises like shoulder flexion, side-lying external rotation, and wall push-ups. Side-lying external rotation, performed with a light dumbbell, helps activate the rotator cuff muscles. Wall push-ups with hands at shoulder height allow patients to engage core stability and shoulder strength while reducing load compared to standard push-ups [38].
- Dual-Task Training for Coordination: Patients perform physical tasks, such as catching a ball, while engaging in a mental task like counting backward or solving simple math problems. For instance, a clinician may toss a ball to the patient at intervals while asking them to count by sevens, enhancing coordination and cognitive processing under simultaneous tasks [39].
- Perturbation Training: In perturbation exercises, the clinician applies gentle, unpredictable forces to the patient’s arm while they hold it in a specific position (e.g., shoulder at 90 degrees of abduction). This helps the patient develop reflexive stability and quick adjustments, essential for joint control in daily and athletic activities (Figure 3).

### 6.8. Criteria to Progress to Phase 5

- The patient consistently performs resistance and strengthening exercises without signs of fatigue, instability, or pain.
- Demonstrates adequate strength and endurance to engage in dual-task and perturbation training with coordination and stability.
- Full participation in daily activities and non-strenuous physical tasks without limitations.
- Sufficient neuromuscular control to handle increased load and dynamic exercises safely.

### 6.9. Phase 5: Advanced Strengthening and Return-to-Sport Phase (Weeks 18–26)

In the final phase, exercises become intensive and mimic real-life or sport-specific movements to ensure the shoulder is prepared for complex physical demands [3,4,21,32,33,34,35,36,37] (Table 5).

**Figure 4:**
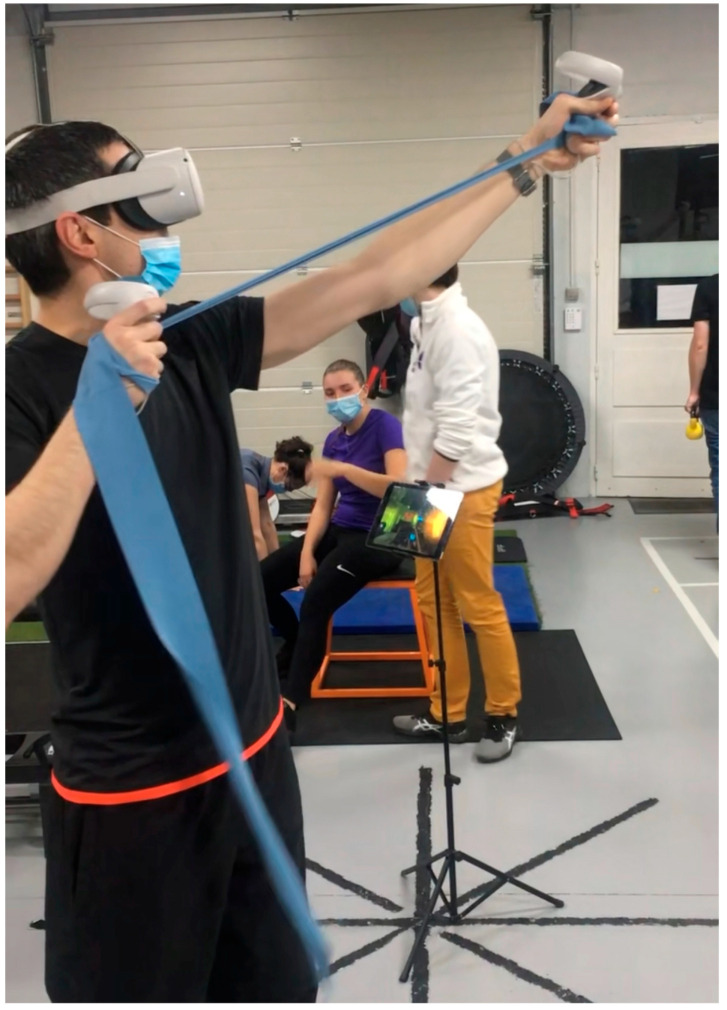
Advanced VR simulations for sport-specific drills.

**Figure 5:**
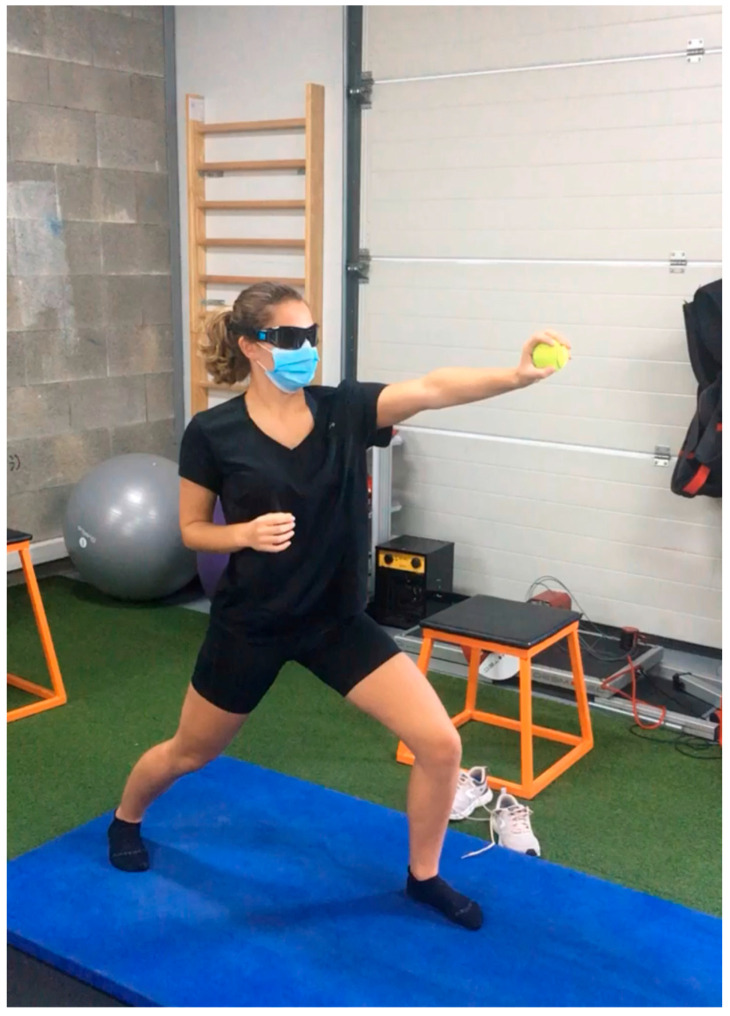
Stroboscopic and dual-task challenges.

- Dynamic Strength Training: Using resistance bands, free weights, and Proprioceptive Neuromuscular Facilitation patterns, clinicians guide patients through complex movements, like diagonal shoulder patterns, that activate multiple muscle groups. Push-up progressions can advance from wall push-ups to floor push-ups, depending on the patient’s strength (Figure 3).
- Advanced VR Simulations for Sport-Specific Drills: For athletes, VR can simulate sport-specific scenarios, like a tennis serve or basketball shot. For example, a tennis player may practice the mechanics of a serve in a VR environment, reacting to virtual cues and simulating real-time decisions. This helps train sport-specific coordination and cognitive engagement (Figure 4).
- Stroboscopic and Dual-Task Challenges: Stroboscopic glasses and dual-task training can be combined for agility and neurocognitive adaptability. For instance, patients can perform a Fitlight reaction drill, using the glasses to create intermittent vision while touching lights that appear randomly. This exercise strengthens focus, reaction speed, and adaptability, key elements for athletic performance (Figure 5).

These phase-specific examples provide clinicians with detailed, actionable steps for each stage of recovery, ensuring that the protocol is adapted to the patient’s progress while targeting both physical and neurocognitive rehabilitation goals.

### 6.10. Criteria to Progress to Unrestricted Activity

- Symmetrical shoulder strength and range of motion compared to the non-injured side.
- Successful execution of sport-specific or work-specific tasks in controlled environments (e.g., VR simulations, agility drills).
- Demonstrates confidence and readiness for unrestricted activity based on clinical evaluation and patient-reported outcomes.
- Absence of pain, instability, or functional limitations during simulated real-life movements or high-intensity tasks.

## 7. Future Perspectives

Our new periodization model—the reverse linear neuro periodization model—is a new model proposed for the long-term rehabilitation needed after an arthroscopic rotator cuff repair. With recent evidence supporting neural contributions to shoulder injuries and the rate of recovery, rehabilitation protocols may benefit from incorporating approaches that target the sensorimotor system. Integrating motor learning principles (external focus and differential learning) and new technologies (virtual reality, lasers, stroboscopic glasses) may bolster current shoulder rehabilitation protocols and improve patient recovery [11,21]. Whether conceptualized and directed by coaches or by themselves, competitive athletes structure their training in a cyclic fashion, enabling themselves to realize their performance capacities and goals best [6,40]. In practical application, sports physical therapists use periodization, a typical example being postoperative “protocols” that serve as rudimentary forms of periodization, albeit implemented over shorter time frames than typically used in preparation for competition. A rotator cuff injury and an arthroscopic rotator cuff repair should not be considered a ‘simple’ musculoskeletal pathology with only local mechanical or motor dysfunctions. Considering the psychological trauma and the reduction in physical capacity, there is a cascade of likely events across the whole spectrum, including neurological insult to the central nervous system and reduction in afferences to the sensorimotor system [6]. Sport rehabilitation specialists should have a basic understanding of periodization theory. Such an understanding can help sports medicine experts interact with the competitive mindset of athletes, their coaches, and their goals better. A basic understanding of these theories and models may help sports rehabilitation specialists to skillfully plan rehabilitation protocols that progress toward realizing the patients’ treatment goals. Such an understanding allows well-informed sports rehabilitation specialists to bridge better the gap between the preparation for competition widely used by coaches and the treatment of injuries that may occur.

To establish the clinical value of the reverse linear neuro-periodization model, we recommend a multi-tiered research agenda: (i) large, multicentre randomised controlled trials comparing the full protocol with standard time-based rehabilitation, powered for return-to-sport and re-tear rates; (ii) factorial or adaptive-platform designs that can disentangle the independent contributions of neurocognitive versus traditional strength components; (iii) 12- to 24-month prospective cohorts that track both objective metrics (strength, proprioception, dual-task cost, re-injury incidence) and patient-reported outcomes—specifically the American Shoulder and Elbow Surgeons score, the Subjective Shoulder Value, and a neurocognitive-confidence questionnaire; (iv) cost-effectiveness and implementation-science studies across high-, middle-, and low-resource clinics to quantify economic and logistical feasibility; and (v) mechanistic sub-studies employing electroencephalography or wearable-sensor-based kinematics to correlate cortical or movement-pattern adaptations with functional recovery. Collectively, these lines of inquiry will provide the empirical foundation needed to validate and refine the proposed model for widespread clinical adoption.

## 8. Conclusions

The integration of the RLNP model in postoperative rehabilitation following arthroscopic rotator cuff repair represents a promising advancement for long-term recovery. This model provides a holistic approach to rehabilitation, enhancing not only physical strength and flexibility but also addressing essential neurocognitive components aimed at improving sensorimotor coordination and brain resilience. By combining progressive strength, power, and motor learning strategies with innovative technologies such as virtual reality and stroboscopic glasses, this model may accelerate recovery times and improve functional outcomes. However, while the model offers encouraging prospects, its clinical effectiveness has yet to be fully demonstrated. The lack of specific empirical studies comparing this model to traditional rehabilitation approaches limits its widespread adoption. To fully validate its potential, future research should focus on large-scale, multicenter randomized controlled trials comparing this model to standardized rehabilitation protocols, measuring outcomes such as return-to-sport rates, re-injury rates, and objective metrics of strength, proprioception, and cognitive responsiveness.

Additionally, long-term prospective studies are needed to assess the impact of the model on neurocognitive rehabilitation and injury prevention. Such studies will provide the empirical evidence necessary to confirm the model’s superiority over conventional approaches and guide clinicians in integrating it into current clinical practice. While the reverse linear neuro periodization model holds significant potential for enhancing postoperative recovery, its integration into clinical protocols will require solid empirical validation to demonstrate its comparative effectiveness. This empirical validation will be critical for advancing clinical practices and making the model applicable on a larger scale across rehabilitation centers.

## Figures and Tables

**Table 1:** Immediate postoperative phase (weeks 1–2).

Objective	Exercises	Description
Protect the repair, reduce pain and inflammation	Passive Range of Motion	Gentle pendulum exercises, assisted shoulder elevation; passive movements only to prevent stiffness and maintain initial joint mobility without stressing the repair.
Neuromuscular re-education	Mental Visualization	Patient visualizes shoulder movements (e.g., flexion, rotation) to engage motor planning and neural pathways, guided by the clinician.
Stimulate early neural adaptation	Cognitive Interference Tasks	Patient responds to auditory cues while focusing on shoulder stability, initiating neural connections without active physical movement.

**Table 2:** Intermediate postoperative phase (weeks 3–6).

Objective	Exercises	Description
Regain controlled mobility	Active-Assisted Movements	Wall slides and shoulder abduction with assistance; patient uses their non-affected arm or a therapist’s support to maintain gentle motion.
Enhance proprioception	Laser Pointer Exercises	Patient traces shapes on a wall using a laser pointer attached to their hand, improving control, proprioception, and spatial awareness.
Improve sensory processing	Stroboscopic Glasses Exercises	With stroboscopic glasses, patient performs shoulder flexion or static holds, adapting to intermittent visual disruption to strengthen reaction time and coordination.

**Table 3:** Active range of motion and strengthening phase (weeks 6–12).

Objective	Exercises	Description
Build active ROM and stability	Active Shoulder Elevation and Rotation	Gradual shoulder movements, such as elevation and rotation, in a supported position (e.g., supine), progressing to seated/standing as patient tolerates.
Introduce gentle resistance	Resistance Band Exercises	Exercises like seated rows and shoulder external rotation with light resistance bands to safely engage stabilizing shoulder muscles.
Enhance hand–eye coordination	VR-Based Neurocognitive Drills	Patient engages with interactive VR scenarios, such as a virtual game requiring small shoulder movements to “catch” or “block” objects, challenging reaction time and control.

Note: ROM; Range of Motion.

**Table 4:** Initial strengthening phase (weeks 12–18).

Objective	Exercises	Description
Increase strength and endurance	Strengthening with Resistance	Progressive resistance added through exercises like wall push-ups, shoulder flexion with dumbbells, and side-lying external rotation to engage and strengthen shoulder muscles.
Enhance cognitive processing	Dual-Task Training	Patient performs a physical task, like catching a ball, while completing a cognitive task, such as counting backward, reinforcing coordination and cognitive focus.
Develop reflexive stability	Perturbation Training	Clinician applies unpredictable forces to the patient’s arm while it is in a static position, training the patient to react and stabilize the shoulder quickly.

**Table 5:** Advanced strengthening and return-to-sport phase (weeks 18–26).

Objective	Exercises	Description
Maximize functional strength	Dynamic Strength Training	Exercises like diagonal shoulder patterns with resistance bands, free weights, and PNF patterns activate multiple muscle groups in functional movements.
Simulate sport-specific movements	Advanced VR Simulations	For athletes, VR simulates a sports environment (e.g., tennis serve or basketball shot), helping the patient practice specific movements and coordination for return to sport.
Enhance reaction and agility	Stroboscopic and Dual-Task Challenges	Combining stroboscopic glasses with dual-task drills like Fitlight exercises (touching lights with intermittent vision), reinforces agility, speed, and cognitive adaptability.

Note: PNF; Proprioceptive Neuromuscular Facilitation, VR; Virtual Reality.

## Data Availability

No new data were created or analyzed in this study.
